# The dual role of type I interferons in bacterial infections: from immune defense to pathogenesis

**DOI:** 10.1128/mbio.01481-25

**Published:** 2025-06-17

**Authors:** Mingjie Qiu, Jiayang Li, Wenqi Wu, Jianan Ren, Xiuwen Wu

**Affiliations:** 1 Research Institute of General Surgery, Jinling Hospital, the Affiliated Hospital of Medical School, Nanjing Medical University 12461, Nanjing, Jiangsu, China; 2 Research Institute of General Surgery, Jinling Hospital, the Affiliated Hospital of Medical School, Nanjing University 12581, Nanjing, Jiangsu, China; The University of Mississippi Medical Center, Jackson, Mississippi, USA

**Keywords:** type I interferon, bacterial infection, host immunity

## Abstract

Type I interferons (IFNs) are crucial components of the human immune system, playing a key role in regulating immune activity. Existing literature has characterized their antiviral and proviral roles in viral contexts. Type I IFNs also exhibit a dual role in bacterial infections, functioning as defenders or disruptors or both based on factors including but not limited to such things as the bacterial species, infection stage, host immune status, and the route and site of infection. This review provides a summary of the signaling pathways associated with type I IFN responses and discusses the complex mechanisms of type I IFNs in bacterial infections. Because type I IFNs provide an important opportunity to develop personalized treatment strategies, which are expected to be transformed into efficient adjuvant therapy for specific infectious diseases, we also discuss the potential for targeting type I IFNs for therapeutic interventions.

## INTRODUCTION

The discovery of interferons (IFNs) can be attributed to the pioneering study conducted by Isaacs and Lindemann in England (1, 2). The term “interferon” refers to a class of secreted proteins originally identified for their ability to interfere with influenza virus replication. Over the past decades, research has revealed the roles of IFNs in a variety of cancers and viral diseases, while recent studies have increasingly uncovered the complex roles of IFNs in bacterial infectious diseases (3–5).

IFNs are categorized into three types according to their structural features, biological functions, and receptor-binding characteristics: type I IFN, type II IFN, and type III IFN. The type I IFN family comprises 14 isoforms of IFN-α as well as IFN-β, IFN-ε, IFN-κ, IFN-ω, and IFN-ζ (6). Among these, IFN-α and IFN-β are the most highly expressed (7). Type II IFN consists solely of IFN-γ, whereas the type III IFN family includes IFN-λ1, IFN-λ2, IFN-λ3, and IFN-λ4 (8).

**Fig 1:**
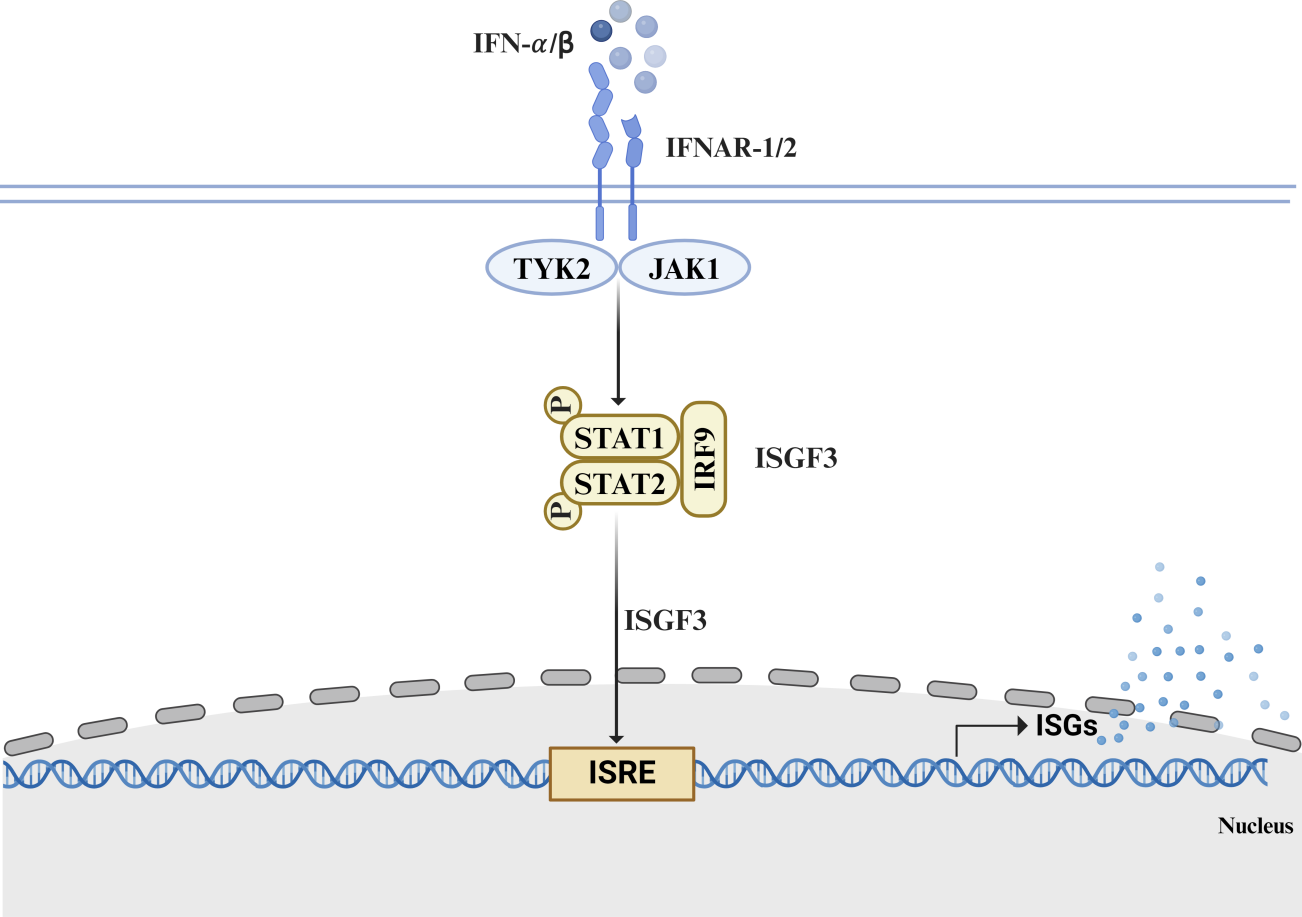
Type I IFN signaling induces ISG expression. Type I IFNs bind to the IFNAR, consisting of the IFNAR1 and IFNAR2 subunits. This interaction activates TYK2 and JAK1, leading to the phosphorylation of STAT1 and STAT2. The phosphorylated STAT1 and STAT2 associate with IRF9 to form the ISGF3 transcriptional complex. ISGF3 translocates to the nucleus and interacts with ISREs in the nucleus, driving the expression of hundreds of ISGs. IFNAR, interferon-α/β receptor; TYK2, tyrosine kinase 2; JAK1, Janus kinase 1; STAT1, signal transducer and activator of transcription 1; IRF9, interferon regulatory factor 9; ISGF3, interferon-stimulated gene factor 3; ISREs, interferon-stimulated response elements; ISGs, interferon-stimulated genes. Figures were created and authorized by BioRender (http://www.biorender.com).

Type I IFNs trigger a series of complex intracellular signaling processes by interacting with specific interferon-α/β receptor (IFNAR), composed of the IFNAR1 and IFNAR2 chains on the cell membrane, thereby exerting biological functions (9). Upon binding to IFNAR, type I IFNs activate tyrosine kinase 2 (TYK2) and Janus kinase 1 (JAK1), which subsequently phosphorylate signal transducer and activator of transcription 1 (STAT1) and STAT2. The STAT1–STAT2 heterodimer associates with interferon regulatory factor 9 (IRF9) to form the interferon-stimulated gene factor 3 (ISGF3) transcription complex (10). ISGF3 is transported to the nucleus, where it interacts with an interferon-responsive element (ISRE) situated within the promoter sequence of interferon-stimulated genes (ISGs), initiating their transcription and exerting anti-infective and immunomodulatory effects (11) (Fig. 1).

**Fig 2:**
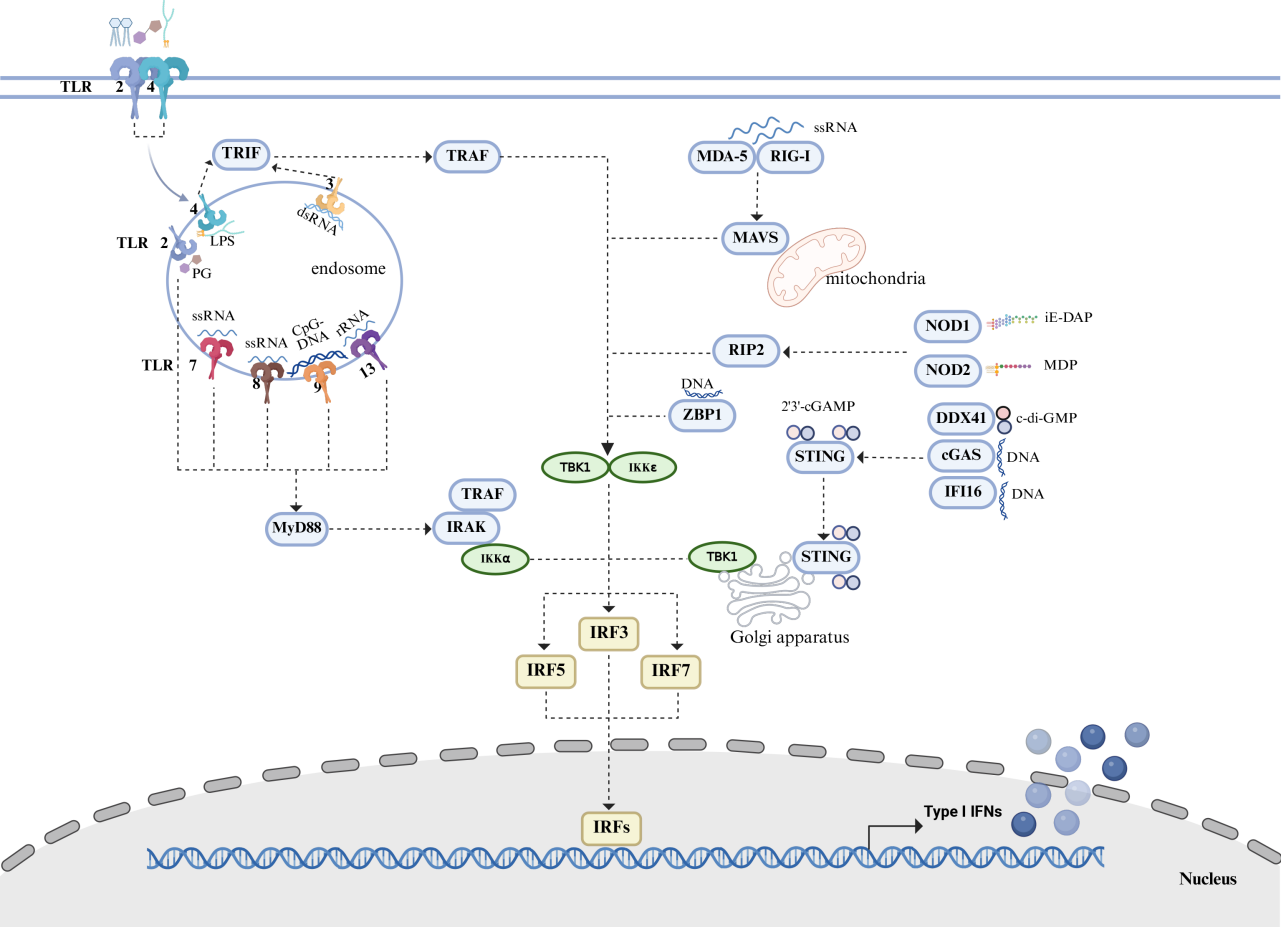
Signaling pathways inducing type I IFNs production in the context of bacterial infections. Recognition of bacterial components by pattern recognition receptors leads to the production of type I IFN. TLR2/7/8/9/13 recognizes DNA or RNA and induces type 1 IFN production through the MyD88-dependent pathway, while TLR3/4 is through the TRIF-dependent pathway. MDA-5 and RIG-I recognize RNA and interact with MAVS, which activates TBK1 and IRF to induce type I IFN production. NOD1 and NOD2 bind the downstream adaptor molecule RIP2 and activate IFN production upon recognizing specific components of bacterial peptidoglycan. DNA sensors cGAS, DDX41, and IFI16 recognize bacterial DNA or cyclic dinucleotide and induce IFN production through the STING-TBK1-IRF3 pathway, respectively. Bacteria-derived 2'3'-cGAMP could also bind STING directly and induce IFN production. ZBP1 is reported to function as a cytosolic receptor sensing *Brucella abortus* infection. TLR, Toll-like receptor; MyD88, myeloid differentiation primary response protein 88; MAVS, mitochondrial antiviral signaling protein; RIG-I, retinoic acid-inducible gene 1; RIP2, receptor interacting protein kinase 2; MDA-5, melanoma differentiation-associated gene 5; NOD, nucleotide-binding oligomerization domain-containing protein; STING, stimulator of interferon genes; TBK1, TANK-binding kinase 1; DDX41, DEAD-box helicase 41; IFI16, interferon gamma-inducible protein 16; ZBP1, Z-DNA binding protein 1; TRAF, TNF receptor-associated factors; TBK1, TANK binding kinase 1; IRF, interferon regulatory factor; 2'3'-cGAMP, 2'3'-cyclic GMP-AMP.

The role of type I IFN varies in bacterial infections, exerting beneficial or detrimental effects, or both. This review summarizes the signaling pathways associated with type I IFN based on the literature (Fig. 2) and further explores its potential as a therapeutic target.

## PATTERN RECOGNITION RECEPTORS INITIATING TYPE I IFN SIGNALING IN RESPONSE TO BACTERIAL INFECTIONS

Type I IFN is triggered when pattern recognition receptors (PRRs) detect pathogen-associated molecular patterns (PAMPs) or damage-associated molecular patterns (DAMPs) (12). PAMPs are evolutionarily conserved, non-specific pathogenic molecular structures that are absent in host cells and essential for pathogen replication and survival. These products encompass endotoxin lipopolysaccharide (LPS), flagellin, lipoteichoic acid, peptidoglycans, and viral nucleic acids like double-stranded RNA or unmethylated CpG DNA (13). DAMPs, on the other hand, are intracellular molecules that are released from damaged or dead cells as a result of trauma or pathogen infection (14).

**Fig 3:**
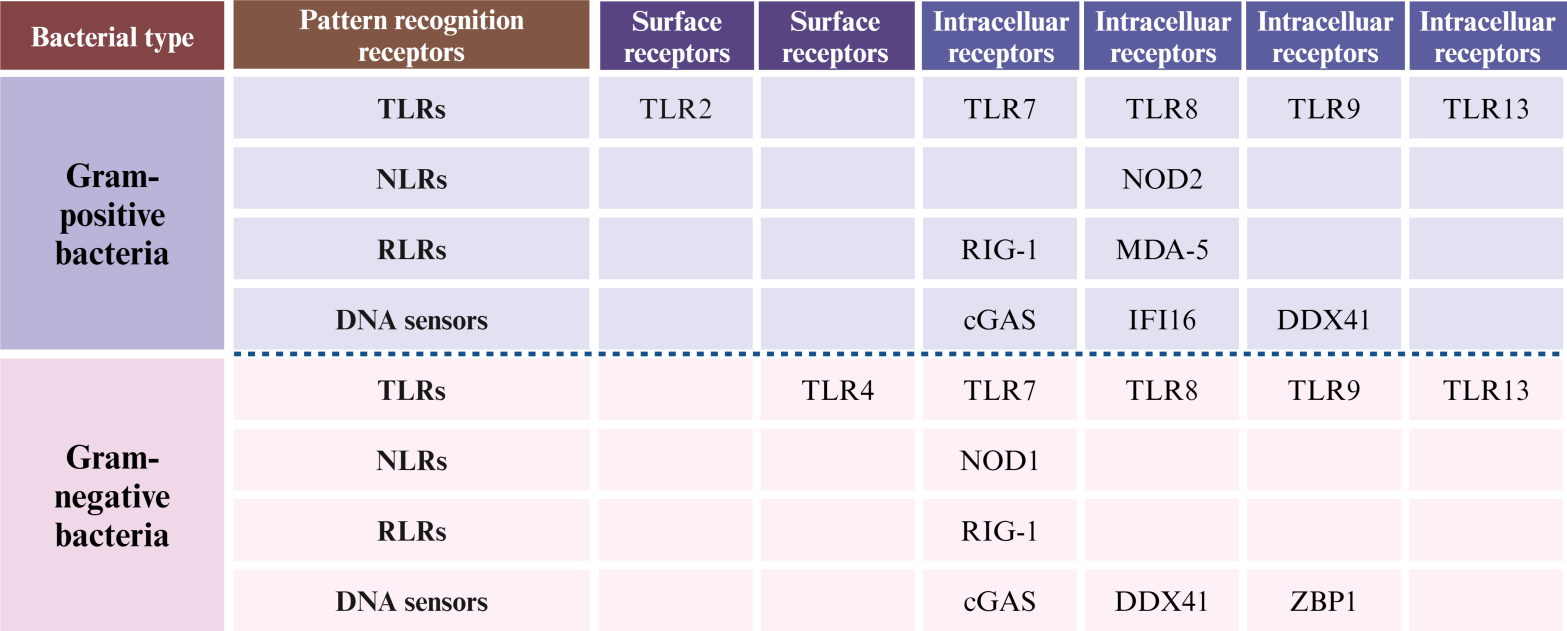
The PRRs involved in the production of type I IFN in Gram-positive and Gram-negative bacterial infections, respectively. The PRRs involved in the production of type I interferon by Gram-positive and Gram-negative bacteria are not exactly the same. Based on the literature reports, the differences and similarities between the two bacterial types are synthesized. The figure is not universally applicable and could be specific to certain types of bacteria.

It is widely acknowledged that the PRR families include Toll-like receptors (TLRs), nucleotide-binding oligomerization domain-like receptors (NLRs), C-type lectin receptors, RIG-I-like receptors (RLRs), AIM2-like receptors, and DNA sensors (15). However, it is important to emphasize that not all PRRs are capable of inducing type I IFN production in response to bacterial infections. Based on current literature, we have summarized PRRs implicated in type I IFN activation in bacterial infections (mainly Gram-positive and Gram-negative bacteria), including TLRs, RLRs, NLRs, and DNA sensors (Fig. 3).

### Toll-like receptors

The TLR was originally discovered in *Drosophila* (16). In 1996, French researcher Jules A. Hoffmann first demonstrated that Toll proteins control the synthesis of drosomycin, a peptide critical for *Drosophila*’s immune response to fungal infections (17). To date, a total of 13 TLRs have been identified: TLR1 to TLR10 are found in humans, while TLR1 to TLR9, along with TLR11, TLR12, and TLR13, are present in mice (18).

TLRs are type I transmembrane glycoproteins consisting of three distinct regions: extracellular, transmembrane, and intracellular. The extracellular region contains leucine-rich repeats that facilitate the recognition of PAMPs, while the transmembrane region and the intracellular Toll-interleukin-1 (IL-1) receptor (TIR) domains are essential for subsequent signal transduction (19). TLR signaling pathways are classified into two categories that primarily induce type I IFNs and inflammatory cytokines: the myeloid differentiation primary response protein 88 (MyD88)-dependent pathway and the MyD88-independent pathway, with the latter also known as the TIR domain-containing adaptor inducing IFN-β (TRIF)-dependent pathway (18).

Different TLRs recognize distinct DAMPS and PAMPS, but not all TLRs induce type I IFN production. Studies have shown that TLR2, TLR3, TLR4, TLR7, TLR8, TLR9, and TLR13 within the TLR family can elicit a type I IFN response upon interaction with their specific ligands after bacterial infections (20–26). For example, LPS is a molecule widely found in the cell wall of Gram-negative bacteria that can trigger a strong immune response. Beutler et al. demonstrated that TLR4 is the key receptor for recognizing LPS. This pioneering research on the molecular mechanisms of innate immunity has had a profound impact on our understanding of the body’s first line of defense against infections (27). TRIF binds to the intracellular portion of TLR4 and activates IRF3 when LPS binds to TLR4, thereby inducing the production of type I IFN (18). In mammalian genomes, CpG sequences are predominantly methylated, whereas in bacterial and viral DNA, they are typically unmethylated. As a result, the host immune system can recognize unmethylated CpG DNA as a signature of exogenous pathogens and trigger an immune response. Specifically, unmethylated CpG DNA is detected by TLR9, which activates the production of type I IFN and pro-inflammatory cytokines (28).

### NOD-like receptors

NLRs are intracellular PRRs consisting of three functional domains: a C-terminal leucine-rich repeat sequence responsible for ligand binding, a central nucleotide-binding domain NACHT required for oligomerization, and an N-terminal protein-protein interaction domain essential for signal transduction (29, 30). NLRs can be further classified into five subfamilies based on the distinct structural domains of their N-terminal effector: the NLRA subfamily, which features the acidic transactivation domain; the NLRB subfamily, which contains the baculoviral IAP repeat structural domain; the NLRC subfamily, which contains the caspase recruitment domain (CARD); the NLRP subfamily, which contains the pyrin domain; and the NLRX subfamily, characterized by the CARD-related X effector domain (31). Upon activation, NLRs mediate innate immune responses through two main mechanisms: the first is that a fraction of NLRs form inflammasomes, thereby triggering inflammation, autophagy, or cell death; the other is by regulating the nuclear factor-kB (NF-kB) and mitogen-activated protein kinase pathways, or by acting as transcriptional regulators in the nucleus to directly regulate gene expression (32). Of the NLRs, only nucleotide-binding oligomerization domain-containing protein 1 (NOD1) and NOD2 from the NLRC family can detect bacterial components and induce type I IFN production. Specifically, NOD1 binds the peptidoglycan gamma-D-glutamyl-endo-diaminoheptanedioic acid (iE-DAP) derived from Gram-negative bacteria and certain Gram-positive bacteria, and NOD2 is able to recognize another constituent of Gram-positive bacterial peptidoglycan, muramyl dipeptide (33–35). In *Helicobacter pylori*-infected human epithelial cells, ligand-bound NOD1 initiates a signaling cascade through the serine-threonine kinase RICK (RIP2). This activation facilitates RICK interacting with TNF receptor-associated factor 3 (TRAF3), subsequently triggering the phosphorylation of TANK-binding kinase 1 (TBK1) and IκB kinase ε (IKKε). The coordinated action of these kinases ultimately activates IRF7 and induces type I IFN (36). Distinctively, in *Mycobacterium tuberculosis*-infected macrophages, the NOD2 pathway engages a molecular mechanism involving RIP2-TBK1-IRF5 to drive type I IFN production (35).

### RIG-I-like receptors

Bacterial RNA can be sensed by RLRs located in the cytoplasm (37). This protein family comprises three members: retinoic acid-inducible gene 1 (RIG-I), melanoma differentiation-associated protein 5 (MDA5), and Laboratory of Genetics and Physiology 2 (LGP2) (38). All RLRs possess a central helicase domain and a carboxy-terminal structural domain that collaboratively function to detect RNA. RIG-I and MDA5 additionally have two CARD structural domains that facilitate downstream signaling. Bacterial RNA can bind to RIG-I and MDA5, thereby inducing type I IFN expression, while LGP2, lacking a CARD, is believed to regulate the activity of RIG-I and MDA5. Recognition and binding of the ligands induce conformational changes in RIG-I and MDA5, which expose their CARDs and facilitate interaction with mitochondrial antiviral signaling proteins (MAVS) (39–41). This interaction results in MAVS aggregation and activation at the mitochondrial membrane. Activated MAVS subsequently recruits and activates the downstream signaling molecules TBK1 and IKKε, which then phosphorylate IRF3 and IRF7 to induce type I IFN production (10).

### DNA sensors

DNA sensors are a class of PRRs that differ in origin, structure, molecular mechanism, and cellular distribution but share a common function to detect intracellular microbial DNA and elicit an innate immune response, which are essential defense against various pathogens (42). DNA sensors include cyclic GMP-AMP synthase (cGAS), interferon gamma-inducible protein 16 (IFI16), absent in melanoma 2 (AIM2), Z-DNA binding protein 1 (ZBP1), DEAD-box helicase 41 (DDX41), and TLR9 (42, 43). Among these, cGAS is the most critical bacterial DNA sensor. Human cGAS can synthesize 2′-3′-cyclic GMP-AMP (2′3′-cGAMP) utilizing ATP and GTP as substrates upon binding with free double-stranded DNA.

Recent studies have revealed that cGAS/DncV-like nucleotidyltransferases (CD-NTases) in bacteria exhibit a striking similarity in the three-dimensional structural conformation of their catalytic domains to metazoan cGAS, suggesting that CD-NTases may represent evolutionary precursors of cGAS (44). Notably, the overall architecture of bacterial and mammalian stimulator of interferon genes (STING) proteins also demonstrates remarkable structural parallels. This functional cGAS-STING pathway in bacteria may serve as a defense against phages (45).

Cyclic dinucleotides (CDNs), such as c-di-AMP, c-di-GMP, and 3′3′-cGAMP, play significant roles in bacterial homeostasis and virulence. STING recognizes these bacterial CDNs as PAMPs, thereby activating the innate immune response (46–48). 2′3′-cGAMP or bacterial sources of CDNs are recognized by STING located in the endoplasmic reticulum (ER), and STING experiences a conformational alteration and relocates from the ER to the Golgi through the endoplasmic reticulum-Golgi intermediate. The recruitment and activation of TBK1 ensue from this pathway; once activated, TBK1 phosphorylates IRF3, which subsequently translocates to the cell nucleus to initiate type I IFN expression (49, 50). Other sensors that are reported to detect bacterial components and produce type I IFN include IFI16, DDX41, and ZBP1 (51–54).

## THE ROLES OF TYPE I IFNS DURING BACTERIAL INFECTIONS

**Fig 4:**
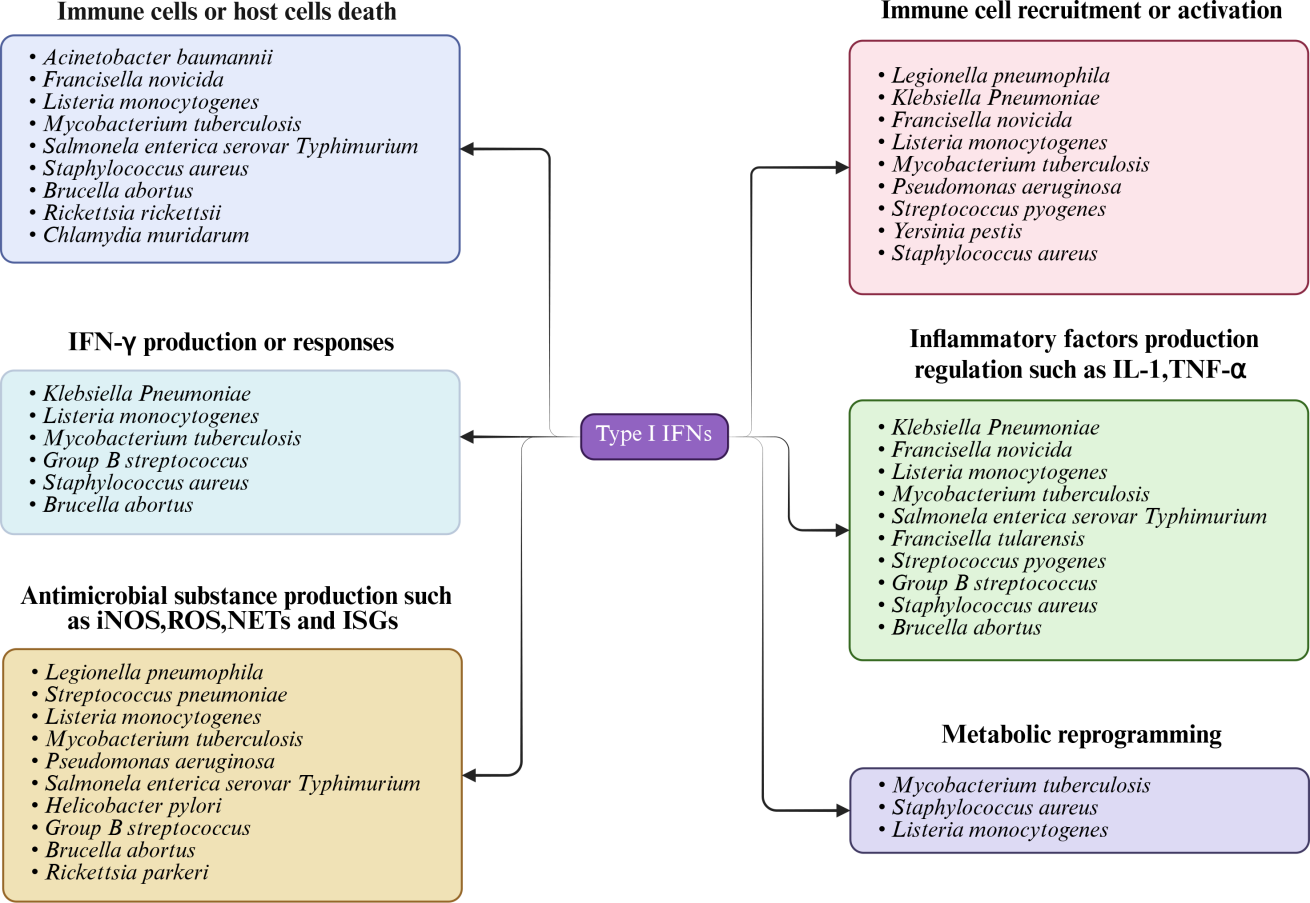
Mechanisms of type I IFNs during bacterial infections. This figure illustrates the mechanisms of type I IFN during bacterial infection, including the induction of immune cell or host cell death, influencing immune cell recruitment and activation, influencing the production of inflammatory factors, antimicrobial substances, and IFN-γ. Studies have included metabolic reprogramming of type I IFN to modulate the inflammatory response during infections with *Listeria monocytogenes, Staphylococcus aureus,* and *Mycobacterium tuberculosis*.

The roles of type I IFNs in bacterial infections are not solely dictated by the pathogen itself but are shaped by the interplay of host-pathogen interactions. Several factors that contribute to these differing outcomes include the type of bacteria, the stage of infection, the immune status of the host, the route or site of infection, and so on. To explore the roles of type I IFNs in bacterial infection, we adopted a classification based on the impact on the host (Table 1). This classification is based on the fact that the roles of IFNs during bacterial infections are regulated by multiple factors, which often lead to different outcomes under various infection contexts and host immune conditions. Therefore, we aim to uncover its complex immune regulatory mechanisms from the host perspective (Fig. 4).

**TABLE 1:** The effects of type I IFN signaling during bacterial infections

Bacterium	Type of bacteria	Location	Route of infection	Effect of type I IFN signaling	Mechanism	Reference
*Acinetobacter baumannii*	Gram(−)	Extracellular	Intranasal	Protective	Type I IFN signaling-mediated modifications of H3K27ac regulated the expression of ZBP1, MLKL, Caspase-11, and GSDMD	(55)
			Intraperitoneal		IFN-inducible protein GBP1 enhanced host resistance to infection by promoting inflammasome activation rather than through direct bacterial killing	(56)
*Legionella pneumophila*	Gram(−)	Intracellular	Intranasal	Protective	Type I IFN relied partly on Irgm1 and other ISGs to mediate antimicrobial effects	(57)
			Intranasal		Type I IFN signaling induced IRG1, catalyzing the broad-spectrum bactericidal metabolite itaconic acid to inhibit LCV in macrophages	(58)
			*In vitro*		IFN-β restricted intracellular replication of *L. pneumophila* in lung epithelial cells	(59)
			*In vitro*		Type I IFN activated M1 macrophages and suppressed the intracellular replication of *L. pneumophila*	(60)
*Streptococcus pneumoniae*	Gram(+)	Extracellular	Intranasal	Protective	Type I IFN signaling protects from lung damage, bacterial dissemination, and AECII death	(61)
			Intranasal		IFN-α increased activation of neutrophils and macrophages, releasing reactive oxygen and nitrogen species and killing bacteria	(62)
			Intranasal		Type I IFN enhanced expression of tight junction proteins and decreased lung permeability to reduce pneumococcal transmigration and invasion	(63)
*Klebsiella pneumoniae*	Gram(−)	Extracellular	Intranasal	Protective	Macrophage-derived type I IFN stimulated NK cell IFN-γ production, IFN-γ enhanced IL-12 secretion, and bacterial killing of macrophages	(64)
			Intraperitoneal		Type I IFN activated MAIT cells, modulated MAIT cell pulmonary location, and enhanced the secretion of IFN-γ and granzyme B	(65)
*Francisella novicida*	Gram(−)	Intracellular	Subcutaneous	Detrimental	Type I IFN induced apoptotic caspases and cell death	(66)
			Intradermal		Type I IFN reduced the number of IL-17A (+) γ δ T cells and neutrophil recruitment	(67)
*Francisella tularensis*	Gram(−)	Intracellular	Intranasal	Detrimental	Type I IFN reduced the number of IL-17A (+) γ δ T cells and neutrophil recruitment	(67)
			*In vitro*		IFN-β suppressed the production of IL-12p40 by bacterial-infected dendritic cells	(68)
*Listeria monocytogenes*	Gram(+)	Intracellular	Intraperitoneal	Dual	Type I IFN enhanced the susceptibility of lymphocytes to apoptosis	(69)
			Intravenous		Type I IFN-mediated IFITM3 suppressed the antibacterial activity of phagocytes and promoted systemic infection	(70)
			Intravenous		USP18 facilitated bacterial replication by inhibiting TNF-α signaling	(71)
			*In vitro*		Bacteria secreted sRNA rli32 resisted hydrogen peroxide and promoted bacterial survival within the cell in a type I IFN-dependent manner	(40)
			Intravenous		STING-dependent type I IFN inhibited CD8+ T cell expansion and its protective immunity to infection	(72)
			Intravenous		Type I IFN impaired T-cell response and compromised protective immunity	(73)
			*In vitro*		Type I IFN production sensitized macrophages to *L. monocytogenes*-induced cell death	(74)
			Intravenous		Type I IFN suppressed IFN-γ expression and subsequent macrophage activation	(75)
			Intravenous		Type I IFN inactivated the ERK pathway and impeded neutrophil aggregation, thereby escaping neutrophil-mediated eradication	(76)
			Intragastric		Gastrointestinal infection-induced type I IFN did not promote susceptibility to bacteria	(77, 78)
*Mycobacterium tuberculosis*	Gram(+)	Intracellular	Aerosol	Detrimental	PARP9 upregulated the type I IFN and increased infection susceptibility	(79)
			Aerosol		UreC interacted with host RUVBL2 to inhibit DNA repair, leading to genomic instability, micronuclei formation, and IFN-β production. IFN-β promoted lipid droplet formation and facilitated *M. tuberculosis* intracellular survival	(80)
			Aerosol		IFN-β inhibited the transition to aerobic glycolysis in inflammatory macrophages while promoting mitochondrial dysfunction and stress	(81)
			Aerosol		Type I IFN promoted bacterial replication and stimulated the generation of DNA-rich extracellular traps, which activated plasmacytoid dendritic cells, enhanced type I IFN production, and impaired responsiveness to IFN-γ	(82)
			*In vitro*		Type I IFN signaling mediated *M. tuberculosis*-induced macrophage death	(83, 84)
			*In vitro*		Type I IFN limited the level of IL-1β mRNA expression	(85)
			Aerosol		IL-1 enhances PGE2 synthesis through cyclooxygenase-2 (COX-2) and inhibits excessive type I IFN production to control infections	(86)
			Aerosol		Type I IFN exacerbated disease by inducing neutrophil-mediated lung inflammation and NETs	(87)
*Pseudomonas aeruginosa*	Gram(−)	Extracellular	Intranasal	Dual	The cGAS-STING-IFN signaling protected mice from *P. aeruginosa*-induced acute pulmonary infection	(88)
			Intratracheal		*P. aeruginosa* triggered IFN-β production to promote intracellular through the cGAS-STING-TBK1 signaling	(89)
					Airway acidification increased activation of the IRF3-IFN-β signaling pathway and impaired host resistance to infection	(90)
			Intratracheal		Type I IFN activated neutrophils and enhanced NETs production, which triggered *P. aeruginosa* biofilm formation and supported its colonization	(91)
*Salmonella enterica* serovar Typhimurium	Gram(−)	Intracellular	Intravenous	Dual	Type I IFN led to the necroptosis of macrophages and enabled *S. Typhimurium* to evade the immune response	(92)
			Intraperitoneal; oral		IFN-β inhibited the *S. Typhimurium*-dependent innate induction of IL-1 family cytokines and neutrophil chemokines	(93)
			*In vitro*		Type I IFN signaling can aggravate mitochondrial damage and cell death by preventing Nrf2-dependent antioxidant responses	(94)
			Intragastrical		*S. Typhimurium* induced a cGAS-STING-dependent type I IFN response in murine macrophages. *Cgas^-/-^* and *Sting^-/-^* mice exhibited higher mortality and higher bacterial loads in the liver and spleen	(95)
*Yersinia pestis*	Gram(−)	Extracellular	Intraperitoneal	Dual	*Ifnar*^-/-^ mice carried more neutrophils and were more resistant to infection than WT mice	(96)
			Intranasal		Type I IFN-induced neutrophil responses protected the lung and caused liver pathology through the TLR7 pathway	(97)
*Helicobacter pylori*	Gram(+)	Extracellular	Oral	Protective	The signaling of NOD1 in gastrointestinal epithelial cells engaged the type I IFN-induced pathway to facilitate host defense mechanisms	(36, 98)
*Streptococcus pyogenes*	Gram(+)	Extracellular	Subcutaneous	Protective	Type I IFN reduced the number of recruited neutrophils at the site of infection and limited inflammatory damage and bacterial spread	(99)
			Subcutaneous		Type I IFN signaling limited the overproduction of IL-1β and prevented hyperinflammation	(25)
Group B *Streptococcus*	Gram(+)	Extracellular	Subcutaneous	Protective	Group B *Streptococcus* induced type I IFNs and mediated host defense through the TLR7-MyD88-IRF1-dependent pathway in conventional dendritic cells	(22)
			Intraperitoneal		Type I IFN increased pro-inflammatory and antimicrobial responses of macrophages	(100)
*Staphylococcus aureus*	Gram(+)	Extracellular	Intranasal/intravenous	Dual	Type I IFN promoted bacterial replication by inhibiting TNF-α signaling	(24, 71)
			Cutaneous		Type I IFN-governed immunometabolic checkpoints coordinated inflammation during infection to enhance host defense while minimizing host damage. Chronic type I IFN expression in skin exacerbated inflammation and promoted bacterial dissemination	(101)
			Cutaneous		*Tlr*^-/−^ mice had reduced interleukin IL-1β production, neutrophil recruitment, and increased bacterial growth. *Sting*^−/−^ mice had the opposite effect, enhancing the ability to limit infection	(102)
			Intranasal		Type I IFN promoted *S. aureus* nasal colonization by inducing macrophage and neutrophil apoptosis	(103)
			Intratracheal		Type I IFN induced the generation of granzyme B by neutrophils and eliminated MRSA	(104)
*Neisseria gonorrhoeae*	Gram(−)	Extracellular	*In vitro*	Detrimental	Type I IFN induced by cGAS-STING and TLR4 signaling pathways controlled the intracellular pool of iron and affected bacterial survival	(21)
*Brucella abortus*	Gram(−)	Intracellular	Intraperitoneal	Dual	*Brucella abortus* DNA induced the production of IFN-β, and Ifnar^−/−^ mice were more resistant to infection	(105)
			Intraperitoneal		Type I IFN signaling restricted the replication of bacteria in macrophages	(106)
			Intratracheal		Sting-/- mice showed increased bacterial load in the lungs, spleen, and liver	(107)
*Rickettsia rickettsii*		Intracellular	*In vitro*	Protective	IFN-β signaling restricted attenuated *R. rickettsii* strain in primary human dermal microvascular endothelial cells	(108)
			*In vitro*		TRIM56-mediated type I IFN production inhibited *R. rickettsii* intracellular replication in HeLa and THP-1 cells	(109)
*Rickettsia parkeri*		Intracellular	Intravenous	Protective	Ifnar^−/−^ mice revealed increased tissue necrosis, leukocyte infiltration, and vascular damage in spleens and livers	(110)
*Chlamydia psittaci*		Intracellular	*In vitro*	Protective	*Chlamydia psittaci* inhibited proliferation through the synergistic action of type I IFN and IL-1β	(111)
*Chlamydia trachomatis*		Intracellular	Intravaginal or transcervical	Protective	The yields of chlamydia in Ifnar^−/−^ mice were higher than those in WT mice	(112)
			*In vitro*		Chlamydia trachomatis synthesized c-di-AMP and induced a protective type I IFN response	(113)
			Intravaginal		IFN-α reduced the growth and infectivity of *Chlamydia trachomatis*	(114)
*Chlamydia muridarum*		Intracellular	Intravaginal	Detrimental	Type I IFN inhibition of CD4+ T cell responses exacerbated genital infection in mice	(115)
			Intranasal		WT mice showed more local macrophage apoptosis, increased bacterial load and weight loss compared with Ifnar^−/−^ mice	(116)

### Protective effects of type I IFNs

Based on the context of infection, programmed cell death may play a protective role. This process limits pathogen proliferation and prevents the spread of infection. Beyond eliminating infected cells by actively inducing cell death, this process can also trigger the host immune response through the release of DAMPs and inflammatory cytokines, thereby promoting more effective pathogen elimination. A study highlighted the critical role of type I IFN-dependent programmed cell death against multidrug-resistant *Acinetobacter baumannii* infection. Type I IFN-mediated pyroptosis and necroptosis through epigenetic regulatory modifications, *Ifnar*^−/−^ mice revealed markedly elevated bacterial burdens in their lungs and spleens versus wild-type (WT) mice. Meanwhile, higher immune cell infiltration and inflammatory cytokine production were detected in the lung tissue of WT mice (55). A recent study further revealed that type I IFN signaling drives Caspase-11-dependent activation of the non-canonical NLRP3 inflammasome, while the IFN-inducible protein GBP1 enhanced host resistance to infection by promoting inflammasome activation rather than through direct bacterial killing (56).

*Legionella pneumophila* is an intracellular organism that is a common cause of pneumonia. Once infected, *L. pneumophila* is phagocytosed by macrophages and forms the legionella-containing vacuole (LCV) for replication within host cells. Previous studies have demonstrated that macrophages infected with *L. pneumophila* produce type I IFN in a STING-IRF3-dependent manner. In addition, two RNA-sensing proteins, RIG-I and MDA5, were reported to be involved in the IFN response to *L. pneumophila* (117). Type I IFN treatment significantly suppressed *L. pneumophila* growth in WT but not in *Ifnar*^−/−^ macrophages. The discovery of how type I IFN inhibits *L. pneumoniae* growth revealed a correlation with macrophage polarization to an inflammatory M1 but not M2 phenotype (60). Type I IFN also stimulated a cell-autonomous defense pathway partly dependent on the antimicrobial effects of Irgm1 and other ISGs. This defensive pathway effectively restricted the bacterial replication within their vacuole, yet it did not impede the formation of the LCV or lysosomal fusion to LCV (57). Further study revealed that type I IFN can modify the protein composition of LCVs by promoting the upregulation of immune response gene 1 (IRG1) within mitochondria. This upregulation fostered a close interaction between IRG1 and the LCV, wherein IRG1 catalyzed the production of itaconic acid, a broad-spectrum antimicrobial metabolite, thereby restricting the replication of *L. pneumophila* within the LCV. This multifaceted response underscored the role of type I IFN in shaping a robust antimicrobial defense by influencing cellular metabolic pathways and enhancing the bactericidal activity within vacuolar compartments (58).

*Streptococcus pneumoniae* secreted hydrogen peroxide, and pneumolysin damaged lung tissue and mitochondria, causing the release of mitochondrial deoxyribonucleic acid and inducing the expression of type I IFN (118, 119). Mice infected with *S. pneumoniae* displayed significant weight loss, pronounced pulmonary inflammation, and a rapid increase in bacterial growth. However, those receiving an adenoviral vector expressing IFN-α demonstrated significantly increased resistance to *S. pneumoniae* infection. The primary mechanism involved the activation of innate immune cells, such as macrophages and neutrophils, which were crucial in the early immune response. Type I IFN boosted the phagocytosis and bacterial killing ability of these cells and promoted the secretion of pro-inflammatory cytokines, thereby effectively limiting the spread of *S. pneumoniae* within the host (62). This contrasted with the suppression of chemokine expression by type I IFN signaling during *Streptococcus pyogenes* infection to reduce neutrophil recruitment and minimize the risk of tissue destruction, highlighting the context-dependent nature of type I IFN effects in different infections (99). The protective role of type I IFN is not only in activating immune cells, but also in preserving the physical barriers that are critical to limiting infection and ensuring the survival of the host. Intranasal infection models of mice lacking the IFNAR1 or treated with neutralizing antibodies against IFNAR1 showed enhanced development of bacteremia. There were two potential mechanisms through which type I IFN regulated pneumococcal invasion: one involved the reduction of lung permeability via the stabilization of tight junction proteins, while the other entailed downregulation of platelet-activating factor receptors, subsequently decreasing lung permeability and thereby limiting bacterial translocation (63). In addition, type I IFN also had a selective protective effect on alveolar epithelial cell type II (AECII) from inflammation-induced cell death, maintaining the integrity of the lung epithelial barrier and reducing tissue damage, inflammation, and bacterial load (61).

Type I IFN signaling critically orchestrated innate immune crosstalk during *Klebsiella pneumoniae* infection. Alveolar macrophages produced type I IFN upon bacterial detection, which directly primed natural killer (NK) cells to secrete IFN-γ. This cytokine reciprocally activated macrophages, amplifying IL-12 production and bactericidal capacity—a self-reinforcing loop essential for pathogen containment. Notably, *Ifnar*^−/−^ mice exhibited defective bacterial clearance, rescued partially by exogenous IFN-γ supplementation (64). Concurrently, type I IFN non-canonically activated mucosal-associated invariant T (MAIT) cells, bypassing MR1-dependent antigen presentation to enhance their secretion of IFN-γ and granzyme B. Type I IFN further directed MAIT cell aggregation around bronchial sites, with IFNAR blockade disrupting this spatial orchestration and compromising local antimicrobial defense (65).

In group A *Streptococcus* cellulitis models, IFN-β production exhibited cell type-specific regulation. In macrophages, IFN-β synthesis required IRF3, STING, and TBK1, but in conventional dendritic cells, IFN-β production was entirely contingent upon IRF5 and MyD88 (99). Despite no bacterial load difference being found between WT and *Ifnar*^−/−^ mice, type I IFN signaling conferred protection by tempering neutrophil infiltration and suppressing IL-1β hyperproduction—a critical brake on immune cell-driven cytokine storms that outweighed direct bactericidal effects (25). In conclusion, type I IFN can exert a significant protective role in diverse bacterial infections by mechanisms such as promoting programmed cell death, enhancing the antimicrobial capability of host immune cells, regulating the inflammatory response and cell death, and safeguarding tissue integrity. However, while programmed cell death can limit bacterial dissemination by eliminating infected cells, excessive programmed cell death may deplete immune populations or release DAMPs that exacerbate inflammation. Similarly, excessive or uncontrolled inflammasome activation may further damage host tissues and form a state of immune dysregulation. Excessive secretion of cytokines can trigger cytokine storm, causing severe damage to host tissues.

### Detrimental effects of type I IFNs

The STING-dependent type I IFN response to *Francisella novicida* required both the cytosolic DNA sensors cGAS and IFI204 (52). Several studies have shown that WT mice infected with *F. novicida* exhibited significantly lower mortality rates than *Ifnar*^−/−^ mice, suggesting that the absence of type I IFN signaling may exert a protective effect (52, 120). A study conducted by Zhu et al. showed that the pathogenic role of type I IFN signaling dominated the protective AIM2 response because type I IFN induced apoptosis in the liver after infection (66). Notably, distinct from the protective role of programmed cell death during *A. baumannii* infection, the apoptotic phenomenon observed in this study demonstrated marked tissue-damaging characteristics. This mechanistic divergence likely stems from pathogen-specific characteristics.

In a mouse model of *Listeria monocytogenes* infection, type I IFN signaling has been demonstrated to enhance the susceptibility of lymphocytes to apoptosis caused by *L. monocytogenes* hemolysin O (LLO). Excessive lymphocyte apoptosis led to an immunosuppressive state and exacerbated infection progression by compromising the host immune responsiveness. *Ifnar*^−/−^ mice exhibited fewer apoptotic lymphocytes and enhanced resistance to *L. monocytogenes* infection compared to WT controls (69). LLO is a pore-forming virulence factor that enables *L. monocytogenes* to escape from the phagolysosome, thereby contributing to listeriosis through membrane perforation (121). Additionally, type I IFN impeded phagosome maturation and the degradation of virulence factors ActA and LLO. This effect was mediated by the induction of the antiviral transmembrane protein IFITM3, which impaired phagosomal function and prevented the hydrolysis of the carriers ActA and LLO, thereby enabling bacterial escape and promoting cell-to-cell transmission. Consequently, bacterial dissemination throughout the host organism was facilitated (70). While previous studies have investigated the mechanism of *L. monocytogenes* macrophage interaction *in vitro*, a recent study indicated that *L. monocytogenes* escaped from Kupffer cells predominantly via LLO-mediated cell membrane perforation rather than through ActA *in vivo*. This study provided a different perspective, exploring the mechanisms by which the host controlled bacterial infection after *L. monocytogenes* escaped from macrophages. It was found that neutrophil swarming effectively cleared escaped *L. monocytogenes* but also resulted in the death of bystander uninfected hepatocytes, thereby limiting *L. monocytogenes* spread. Meanwhile, *L. monocytogenes* employed type I IFN to suppress neutrophil aggregation by antagonizing ERK signaling and evading immune surveillance (76). These studies highlighted the distinctions between *in vivo* and *in vitro* bacterial infections and emphasized the complexity of diverse escape strategies based on distinct immune cells and infection context.

Additionally, *L. monocytogenes* can promote infection through other type I IFN-dependent mechanisms. Ubiquitin-specific peptidase 18 (USP18) is an ISG that exerts an adverse effect during *L. monocytogenes* infection. USP18 deficiency in CD11c-Cre+ cells significantly reduced mortality in lethal infection models. The negative effect of USP18 was independent of isopeptidase activity or effects on cytokine production but rather by suppressing TNF-α signaling to facilitate bacterial replication (71). The secreted small non-coding RNA rli32 induced IFN-β expression via the RIG-I pathway and resisted hydrogen peroxide. In contrast, mutant strains lacking rli32 expression showed restricted growth in macrophages (40). *L. monocytogenes* also released an RNA-binding protein, Zea, alongside RNA into the cytoplasm, forming ribonucleoprotein complexes that activated RIG-I and amplified type I IFN responses, thereby creating a self-reinforcing loop favoring bacterial persistence (122). Interestingly, type I IFN played a protective role in the host when infected with *L. monocytogenes* via the gastrointestinal route (77). This finding differed from that for intravenous or intra-abdominal infections, suggesting that the adverse or beneficial effects of IFN may vary according to the route of infection.

Type I IFN signaling exerted broad immunosuppressive effects across bacterial infections by targeting key cytokines. In γδ T cells, type I IFN signaling suppressed protective IL-17A/F production by regulating the transcription factor RORγt, as *Ifnar*^−/−^ mice exhibited elevated IL-17A/F levels and enhanced resistance against *Francisella tularensis*, *F. novicida*, and *L. monocytogenes* (67). Following infection with *F. tularensis*, dendritic cells produced IFN-β directly inhibited IL-12p40 expression**—**a critical cytokine subunit required for Th1 differentiation and NK cell activation. By silencing IL-12p40, type I IFN disrupted dendritic cell-driven adaptive immunity, creating an immunosuppressive environment that facilitated bacterial immune evasion and systemic dissemination (68). Notably, this IFN-mediated cytokine suppression extended to tuberculosis pathogenesis: type I IFN can directly block the transcription of pro-IL-1β, thereby facilitating the *M. tuberculosis* evasion of host immune surveillance (85). Conversely, IL-1 conferred host resistance through the induction of PGE2 that restricted the excessive production of type I IFN (86).

The interaction between *M. tuberculosis* and the host DNA damage repair system revealed the unique nature of type I IFN signaling in the progression of tuberculosis. Researchers described a type I IFN-dependent intracellular survival strategy related to macrophage DNA repair employed by *M. tuberculosis*. Specifically, they found that *M. tuberculosis* secreted urease C (UreC), which interacted with the host RuvB-like protein 2 (RUVBL2) to inhibit DNA repair by blocking the formation of DNA damage repair complexes. This inhibition triggered genomic instability and micronucleus formation to activate IFN-β production through the cGAS-STING-IRF3 pathway. Although this process was an inherent host response to DNA damage, it was reversely hijacked by the *M. tuberculosis*—UreC-mediated IFN-β upregulated scavenger receptor A1 (SR-A1) and promoted the formation of lipid droplets, providing a lipid nutrient niche for bacterial replication (80). On the other hand, in *Parp9^−^*^/−^ mice, overexpression of type I IFN caused by DNA repair defects can activate the complement and coagulation cascade and inflammatory pathways, thereby increasing disease severity. Blocking type I IFN signaling reversed the heightened susceptibility to *M. tuberculosis* observed in *Parp9^−^*^/−^ mice (79). These findings suggested that *M. tuberculosis* can turn the host DNA repair system into a springboard for autoimmune escape and subvert it into a disease-promoting agent through type I IFN responses.

*L. monocytogenes* infection induced host hepatic metabolic remodeling, such as fatty acid oxidation (FAO) and oxidative phosphorylation, which was partially dependent on the type I IFN signaling pathway. Type I IFN exacerbated damage by promoting FAO, and inhibition of FAO significantly reduced bacterial load in WT mice (123). Recently, the core strategy of metabolic reprogramming by *M. tuberculosis* through type I IFN signaling has also been elucidated. *M. tuberculosis* infection suppressed macrophage glycolysis, a process further exacerbated by type I IFN signaling, which forced metabolic reliance on mitochondrial oxidative phosphorylation. This shift induced an energy crisis, crippling bactericidal functions. Moreover, type I IFN triggered mitochondrial stress characterized by collapsed membrane potential and reactive oxygen species overaccumulation. The resulting energy crisis-oxidative damage axis created a vicious cycle that culminated in metabolic paralysis and mitochondrial dysfunction, rendering macrophages incapable of containing intracellular bacterial replication (81).

In contrast to the antibacterial effects of neutrophil extracellular traps (NETs) on some bacteria, *M. tuberculosis* is capable of utilizing NETs-induced responses to facilitate disease progression. Type I IFN promoted NET generation, which activated plasmacytoid dendritic cells to amplify type I IFN production, creating a self-reinforcing loop. This positive feedback loop impaired responsiveness to IFN-γ, driving inflammation out of control and exacerbating bacterial proliferation (82). A recent study has demonstrated that NETs release mediated by neutrophil type I IFN signaling promoted *M. tuberculosis* replication and was associated with necrosis and the formation of caseum (124). In addition, *M. tuberculosis* recruited immature, functionally impaired neutrophils through a type I IFN-dependent mechanism, promoting bacterial survival, exacerbating epithelial damage, and inhibiting the production of surfactant (125). These studies collectively elucidated the early mechanisms underlying type I IFN susceptibility to *M. tuberculosis* and its correlation with subsequent tissue damage (82, 87, 124). Exogenously added IFN-α and IFN-β significantly exacerbated the death of *M. tuberculosis*-infected human monocyte-derived macrophages (83). Zhang et al. also identified type I IFN signaling as a crucial modulator of *M. tuberculosis*-induced macrophage death in mice through CRISPR-Cas9 screening. Nevertheless, the death of macrophages in the study was not characterized by apoptosis, necroptosis, pyroptosis, autophagy, or other features of cell death. This suggested that there may be a novel mechanism by which type I IFN induces macrophage death (84). The follow-up study demonstrated that IRG1 was significantly upregulated and bound to HSP70 in macrophages, a protein that stabilizes lysosomes. IRG1 promoted the degradation of HSP70, weakening the stability of the lysosomal membrane, ultimately increasing lysosomal membrane permeability and leading to lysosome-dependent cell death (126).

### Dual effects of type I IFNs

*Pseudomonas aeruginosa* triggered IFN-β production through the cGAS-STING-TBK1 pathway. In studies, *P. aeruginosa*-infected *Cgas*^−/−^ and *Sting*^−/−^ mice exhibited increased lethality and bacterial load compared to WT controls, underscoring the critical role of type I IFN in restricting *P. aeruginosa* infection (88). The above conclusions were based on the background that *P. aeruginosa* was an extracellular bacterium. In recent years, there has been evidence that *P. aeruginosa* can reside in host cells. Type I IFN has been shown to promote intracellular survival of *P. aeruginosa* in contrast to the extracellular situation. IFN-β pretreatment enhanced bacterial survival within macrophages by suppressing bactericidal pathways, while *Ifnb*^−/−^ mice exhibited reduced lung injury and bacterial loads. The study also proved that IL-1 acts as a negative regulator of IFN-β production during *P. aeruginosa* infection (89). Liu et al. similarly demonstrated that the intracellular survival of *P. aeruginosa* was significantly higher in the IFN-β pretreated group. They further observed that the acidic airway microenvironment promoted the release of *P. aeruginosa* outer membrane vesicles (PA_OMVs), which enhanced *P. aeruginosa* or PA_OMVs-induced IRF3 phosphorylation and increased IFN-β production. This cascade ultimately impaired host defenses against *P. aeruginosa* lung infection (90). Biofilm is a key factor in the invasion and persistence of *P. aeruginosa*. Type I IFN potentiates NETs release, thereby facilitating biofilm formation and lung colonization. This process was associated with a significantly elevated bacterial load and extensive lung tissue damage (91).

During *Yersinia pestis* infection, the atypical MyD88-independent TLR7 pathway stimulated the production of type I IFN, which resulted in neutrophil depletion and impaired the host’s ability to clear bacteria. In pneumonic plague, the response helped protect the lungs but simultaneously worsened liver pathology. This was because the reduction in neutrophils alleviated inflammatory damage in lung tissue while simultaneously increasing the bacterial load in the liver, resulting in distinct patterns of organ damage (97). For the affected individuals, *Ifnar*^−/−^ and *Tlr7*^−/−^ mice were more resistant to disease (96, 97).

During *Salmonella typhimurium* infection, both protective and detrimental roles of type I IFN have been observed. A recent study revealed that beyond the TLR4-dependent response, *S. Typhimurium* can trigger mitochondrial DNA leakage, activating the cGAS-STING-IFN pathway. *Cgas*^−/−^ and *Sting*^−/−^ mice were more susceptible to *S. Typhimurium* infection and exhibited higher bacterial loads in the liver and spleen, suggesting a critical role for the cGAS-STING-dependent type I IFN response in host defense against *S. Typhimurium* infection. The findings offered some evidence that type I IFN may have a protective effect on the host, although further investigation was required to confirm this (95). Host survival improved in the absence of type I IFN signaling, particularly in *Ifnar*^−/−^ mice, as type I IFN induced macrophage necroptosis via the RIPK3 signaling pathway. This form of cell death releases intracellular *S. Typhimurium*, exacerbating infection spread and inflammation (92). The downstream mechanism of type I IFN has been explored. In infected macrophages, type I IFN induced RIP3-mediated Pgam5 expression, which interacted with Nrf2 through Keap1 and repressed the transcription of Nrf2-dependent antioxidative genes. Meanwhile, bacterial-induced oxidative stress degraded p62 via mitophagy, and reduced p62 levels compromised the interaction of p62 with Keap1 and diminished Nrf2-dependent antioxidative responses to infection, leading to macrophage death (94). Additionally, another study suggested that type I IFN may regulate *S. Typhimurium*-induced macrophage death through caspase-11 (127). IFN-β also selectively suppressed expression of IL-1 family cytokines and neutrophil chemokines, impairing antibacterial innate responses independently of its pro-death function (93).

The *Staphylococcus aureus* strain Newman exploited type I IFN signaling to induce apoptosis in neutrophils and macrophages within nasal tissues, impairing mucosal immunity to facilitate bacterial persistence (103). In pneumonia models, bacterial infection activated type I IFN signaling via TLR9 in dendritic cells. Notably, *Ifnar*^−/−^ and *Tlr9*^−/−^ mice exhibited enhanced protection against MRSA infection compared to their WT counterparts. This protective effect was attributed to a reduction in TNF-α levels, a mechanism shared with ISG USP18 (71), rather than differences in neutrophil recruitment to the airways (24). Strikingly, dendritic cells produced type I IFN also promoted neutrophil granzyme B-dependent MRSA clearance under IL-21 signaling blockade, underscoring its functional plasticity shaped by host immune status (104).

Type I IFN precisely coordinated the inflammatory response of macrophages in MRSA infection through temporal dynamic regulation of immune metabolic checkpoints. During the early stage of infection, TLR signaling enhanced mitochondrial respiration, while the subsequent staggered TLR and IFN signaling coordinated to trigger an immune metabolic checkpoint by inducing iNOS expression, inhibiting oxidative phosphorylation (OXPHOS), and shifting metabolism toward glycolysis. This disruption of OXPHOS biased macrophages toward producing type I IFN instead of IL-1β secretion, resulting in a pro-inflammatory state. However, with the acceleration of glycolysis and the accumulation of lactate, iNOS expression was suppressed through histone lactylation, which negatively inhibited IFN-β production, forming a self-limiting loop to balance inflammatory damage (101). Under chronic type I IFN exposure, this loop was disrupted: sustained iNOS expression led to excessive NO accumulation, while lactate production was impaired, ultimately leading to vascular disease, tissue repair disorders, and pulmonary bacterial spread (101). This finding may provide a theoretical basis for targeting type I IFN signaling for the treatment of MRSA infection: selective intervention based on the stage of infection.

## POTENTIAL TREATMENT OF BACTERIAL INFECTIONS BASED ON TYPE I IFN

In the 1970s, American scientist Sidney Pestka and his research team successfully cloned the human IFN gene, enabling the synthesis of recombinant interferon for medical research. This breakthrough facilitated the development of IFN-based drugs and their widespread clinical application. IFNs have been used to treat a variety of diseases, particularly viral infections, autoimmune disorders, and certain cancers. For instance, IFN-α was used for chronic hepatitis treatment, and IFN-β was used to treat multiple sclerosis (128–131). Prior to the advent of targeted therapies like tyrosine kinase inhibitors, IFN-α was employed in the treatment of chronic myeloid leukemia (132). Additionally, IFN-α has been utilized to prevent relapse after melanoma surgery (133–135).

The role of type I IFN in bacterial infections is complex and controversial. Current studies have mostly been conducted on animal models to explore the potential therapeutic interventions of type I IFN in bacterial infections, although there is still a considerable distance to cover before clinical application. For example, interleukin-1 (IL-1) promoted prostaglandin E2 (PGE2) synthesis via cyclooxygenase-2 (COX-2) to regulate excessive type I IFN production and control bacterial infections. A study demonstrated that the clinically approved drug zileuton, a 5-lipoxygenase inhibitor, competitively interacted with COX-2 to elevate PGE2 levels, thereby preventing acute mortality in *M. tuberculosis*-infected mice. This approach provided a promising avenue for antibiotic replacement or adjunctive therapies aimed at improving infection outcomes (86). Mice treated with an adenoviral vector expressing IFN-α, either before or after *S. pneumoniae* infection, exhibited swift and efficient regulation of bacterial proliferation and pulmonary inflammation, resulting in improved clinical outcomes (62). There were also case reports of IFN-α or IFN-β being used as adjunctive therapies to treat IFNGR-deficient patients, who were highly susceptible to mycobacterial infections, with some showing symptom improvement following treatment, indicating the potential of its clinical application (136, 137).

## CONCLUSION

Type I IFNs play a crucial role in regulating host immunity. Distinct from their well-characterized antiviral functions, their effects on bacterial infections are complex and context-dependent, varying with factors such as the bacterial species, location of infection, stage of infection, and host immune status. Over the past decades, significant progress has been made in understanding the effects of type I IFN in bacterial infections. Studies have elucidated key signaling pathways that regulate type I IFN production and identified specific mechanisms through which type I IFN modulates bacterial infections, including inducing cell death, regulating immune cell recruitment, modulating proinflammatory cytokine expression, and enhancing autonomous immunity (55, 57, 60, 65). These mechanisms have beneficial or harmful effects on the host based on the different infectious contexts and environments. Recent research further highlighted that the role of type I IFN extended beyond direct antibacterial effects and immune regulatory functions, profoundly influencing host defense by modulating immune metabolism (81, 101). This regulatory mechanism may offer a promising avenue for the development of therapeutic strategies targeting type I IFN metabolic pathways.

Despite its widespread application in the treatment of viral infections and cancer, the use of type I IFN in bacterial infections faces numerous challenges. Chief among these is its dual role as both a protector and a potential injury contributor to the host. This dual effect presents a significant clinical challenge: how can we harness its protective properties while mitigating the risks of exacerbated inflammation or tissue damage? Furthermore, the response to type I IFN varies across bacterial species, necessitating pathogen-specific therapeutic strategies. Patient factors, including immune status and genetic background, further complicate its clinical application. Taking these into account, the clinical application of type I IFN essentially demands that we adopt a precision therapy strategy, namely, formulating individualized treatment plans based on patient-specific factors such as the type of immune deficiency, pathogen characteristics, and infection stage. For example, in IFNGR-deficient patients combined with mycobacterial infection, exogenous type I IFN played a therapeutic role by compensating for the missing IFN-γ signaling pathway, which represented a classic example of precisely identifying pathological mechanism defects to guide type I IFN use (136). These findings suggested that type I IFN could serve as a novel therapeutic avenue distinct from traditional antibiotics. To sum up, the dual role of type I IFN in bacterial infections reminds us that the implementation of precision medicine is essential for the development of IFN-based therapeutic strategies. Future research should focus on exploring the cellular and molecular basis of its regulatory mechanism and verify its feasibility as a therapeutic target for bacterial infections through more clinical trials. Combined with the pros and cons, while the application of type I IFN in the treatment of bacterial infections remains in its infancy, it holds significant promise for future therapeutic strategies.
